# Influence of physician and consumer demographics on AI-use penalties in primary care: a vignette-based study

**DOI:** 10.1038/s41746-026-03361-3

**Published:** 2026-10-01

**Authors:** Moritz Reis, Jungmin Choi, Yeun Joon Kim, Yingyue Luna Luan, Pengzhao Lyu, Florian Reis

**Affiliations:** 1 Institute of Psychology, University of Wuerzburg, Wuerzburg, Germany; 2 Institute of Metabolic Science, School of Clinical Medicine, University of Cambridge, Cambridge, UK; 3 Cambridge Judge Business School, University of Cambridge, Cambridge, Cambridge, UK; 4 UQ Business School, The University of Queensland, Brisbane, QLD Australia; 5 Institute of Medical Informatics, Charité – Corporate Member of Freie Universität Berlin and Humboldt-Universität zu Berlin, Universitätsmedizin Berlin, Berlin, Germany

**Keywords:** Health care, Mathematics and computing, Medical research

## Abstract

We investigated how the “AI-use penalty”— physicians disclosing AI use being perceived more negatively—is affected by physician and consumer demographics in primary care settings. Participants (*N* = 1030) assessed fictitious family doctor advertisements varying in AI-use and demographics. Physicians stating that they use (vs. never use) AI were rated as less warm and competent, with reduced appointment willingness. Effects were stronger among younger participants but largely independent of physician demographics.

## Introduction

As artificial intelligence (AI) becomes increasingly integrated into healthcare, its use may influence not only physicians’ clinical practice but also how consumers perceive physicians who use AI. In consumer-facing contexts, where consumers often have limited direct information about a physician’s ability or intentions, AI-use disclosure may function as a salient cue in social evaluations. Consistent with cue-based accounts of trustworthiness, such as the *trustworthiness assessment model* (TrAM^1^), consumers may rely on this cue in ways that lead them to evaluate physicians who disclose AI use less favorably than otherwise comparable physicians who do not. We refer to this pattern as an “AI-use penalty”. Prior work supports this assumption: merely believing AI is involved can undermine trust in medical information^2^, and physicians who disclose AI use are perceived as less competent by both peers^3^ and the public^4,5^.

However, it remains unclear whether this AI-use penalty operates uniformly regardless of physician and consumer demographics, or whether its magnitude varies as a function of these characteristics. TrAM suggests that trustworthiness evaluations are informed by available cues, which, in the present context, include both AI disclosure and physician demographics. This question is important because, even in the absence of AI, consumer evaluations vary systematically by physician ethnicity or race^6^, gender^7^, and age^8^. Beyond cues associated with the physician as the target of evaluation, characteristics of the perceiver may also influence social evaluations. That is, consumers may interpret the same cues differently, as research shows that attitudes toward AI in healthcare differ between women and men^9^ and between Black and White individuals^10^.

To test whether consumer reactions to physicians’ AI-use are shaped by physicians’ and consumers’ characteristics, we conducted a preregistered experiment with a large US-based sample (*N* = 1,030; recruited via Prolific), stratified by age, gender, and race to approximate the composition of the US adult population. Participants were shown fictitious advertisements for family doctors which they should evaluate as part of a supposed market research study. Within these evaluations, we focused on perceived competence and warmth, two fundamental dimensions of social perception that reflect judgments about a person’s ability and intentions^11^. These dimensions are particularly relevant in medical settings, where consumers must assess both whether a physician is capable and whether they act in the consumers’ best interest. In addition, we examined behavioral intentions, such as willingness to make an appointment, to assess downstream consequences of these perceptions. To test the influence of physician and consumer demographics, we assessed participants’ demographics (age, gender, race) and manipulated physicians’ gender (female vs. male), age (relatively younger vs. older), and race (White vs. Black vs. Asian). Furthermore, we varied between-subjects whether the advertisement stated that the doctor uses AI (vs. never uses AI) for diagnostics and therapy.

**Fig. 1:**
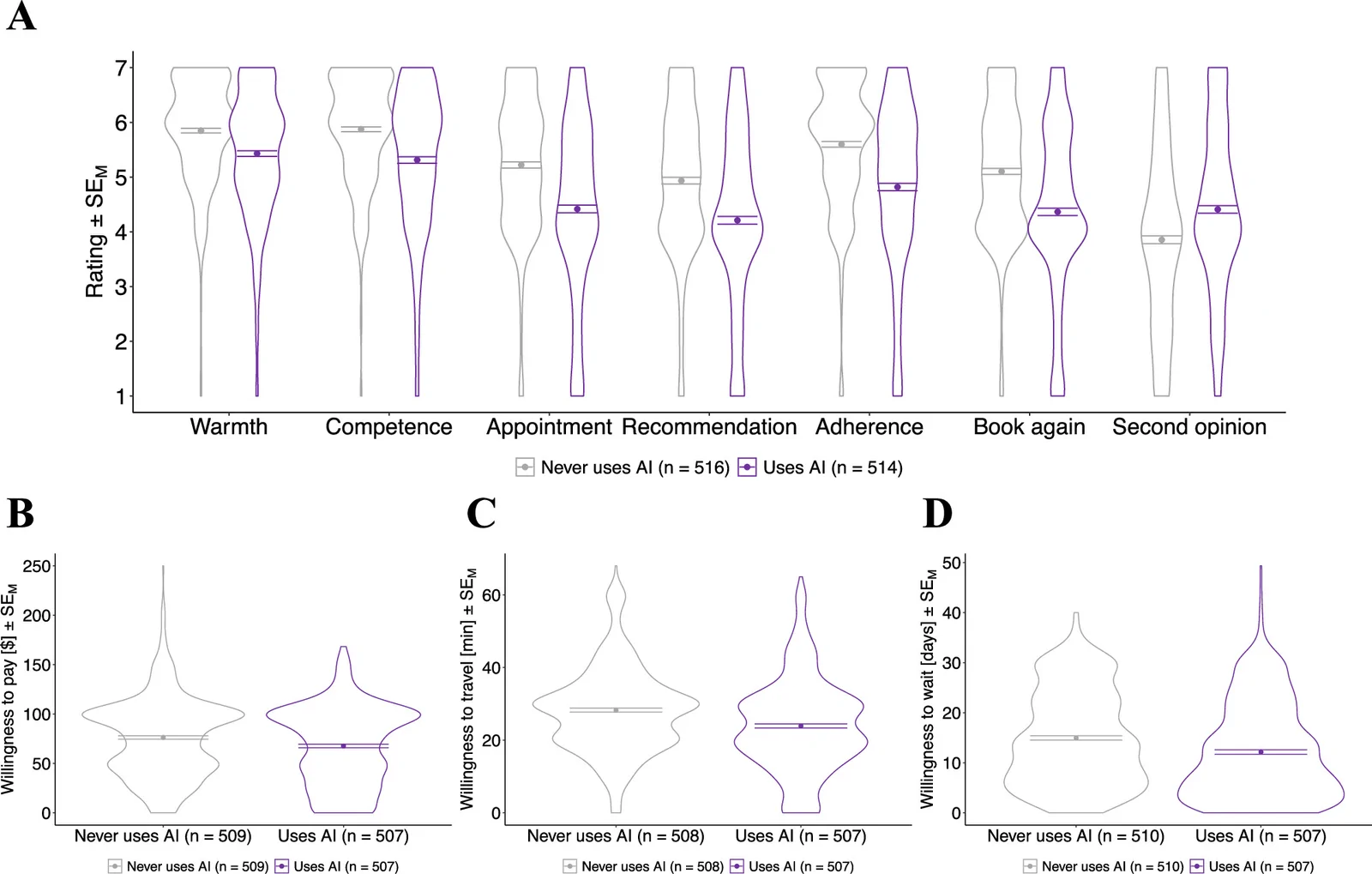
Key results. Physicians who state the use of AI are perceived significantly more negative across all characteristics (**A**). Furthermore, participants would pay less money (**B**), travel less far (**C**), and wait less long (**D**) for an appointment with an AI-using doctor. Error bars show standard errors of the individual means.

Descriptive results for all experimental conditions can be found in Tables S1–S4 in the supplement. Our preregistered analyses showed that participants exhibited a clear aversion toward physicians stating to use (vs. never use) AI (see Fig. 1). Physicians stating AI-use were evaluated as significantly less warm, *F*(1, 1022) = 38.51, *p* < 0.001, *η*_*p*_^2^ = 0.04, and less competent, *F*(1, 1022) = 57.30, *p* < 0.001, *η*_*p*_^2^ = 0.05. In the AI-use condition, participants were also significantly less willing to make an appointment, *F*(1, 1022) = 79.40, *p* < 0.001, *η*_*p*_^2^ = 0.07, to recommend this doctor to others, *F*(1, 1022) = 60.26, *p* < 0.001, *η*_*p*_^2^ = 0.06, to adhere to this doctor’s treatment plan, *F*(1, 1022) = 82.84, *p* < 0.001, *η*_*p*_^2^ = 0.07, or to book with this doctor again, *F*(1, 1022) = 71.69, *p* < 0.001, *η*_*p*_^2^ = 0.07. Similarly, in the AI-use condition, participants were significantly more likely to request a second opinion from another doctor, *F*(1, 1022) = 30.87, *p* < 0.001, *η*_*p*_^2^ = 0.03. Finally, participants indicated that they would travel less far, *F*(1, 1007) = 31.71, *p* < 0.001, *η*_*p*_^2^ = 0.03, pay less money, *F*(1, 1008) = 11.76, *p* = 0.001, *η*_*p*_^2^ = 0.01, and wait less long for an appointment with a doctor who discloses AI-use, *F*(1, 1009) = 21.59, *p* < 0.001, *η*_*p*_^2^ = 0.02. None of the effects significantly interacted with physician characteristics (age, gender, race) after applying Benjamini-Hochberg correction, *F*s ≤ 3.61, *p*s ≥ 0.028 (smallest unadjusted *p*-value; adjusted threshold for statistical significance for this comparison is *p* = 0.005).

**Fig. 2:**
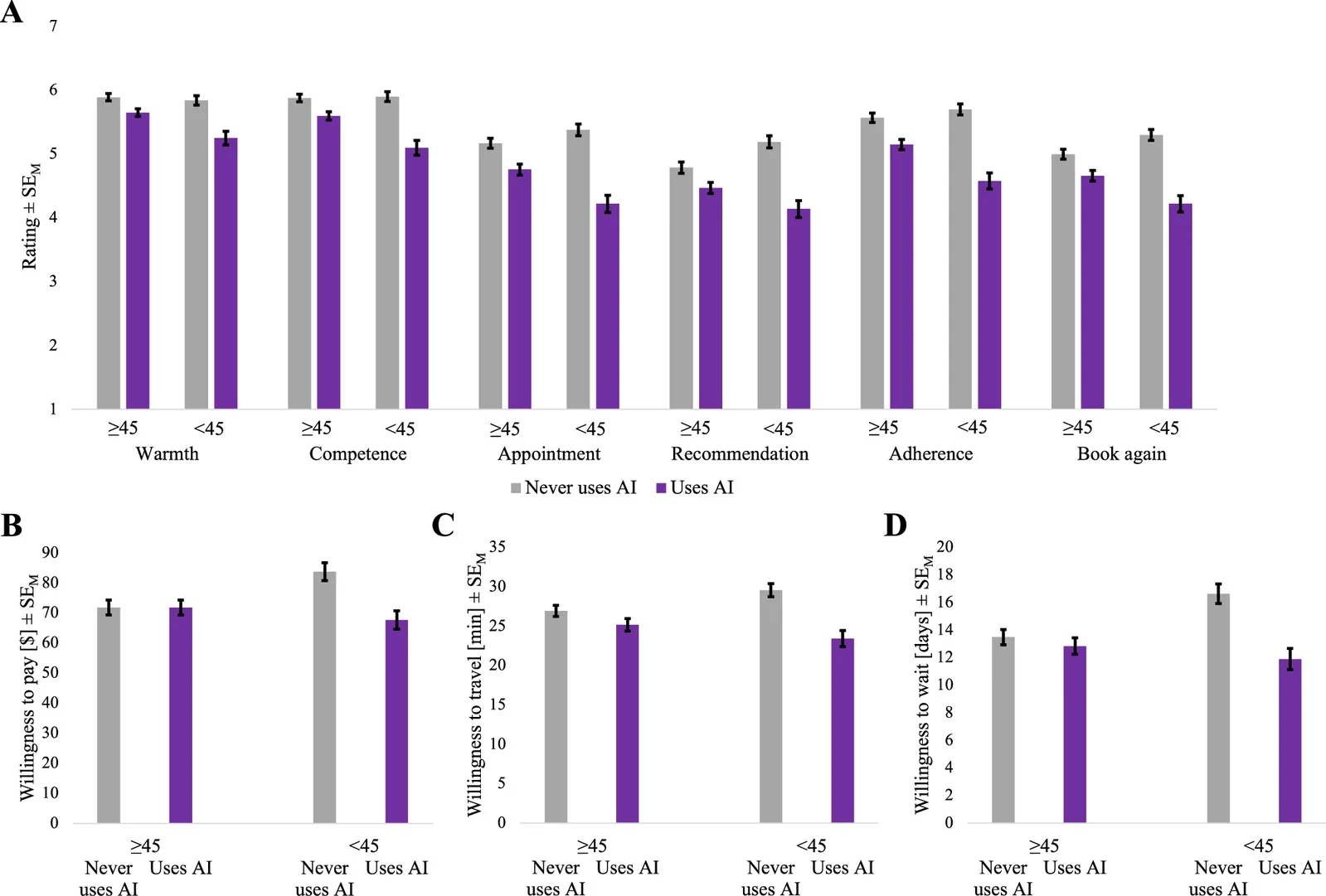
Influence of consumer age. AI-use penalties for most rating dimensions were more pronounced for participants below 45 years (**A**). Along the same lines, for younger participants, AI-use had stronger effects on the willingness to pay (**B**), travel (**C**) and wait (**D**) for an appointment. Error bars show standard errors of the individual means.

**Fig. 3:**
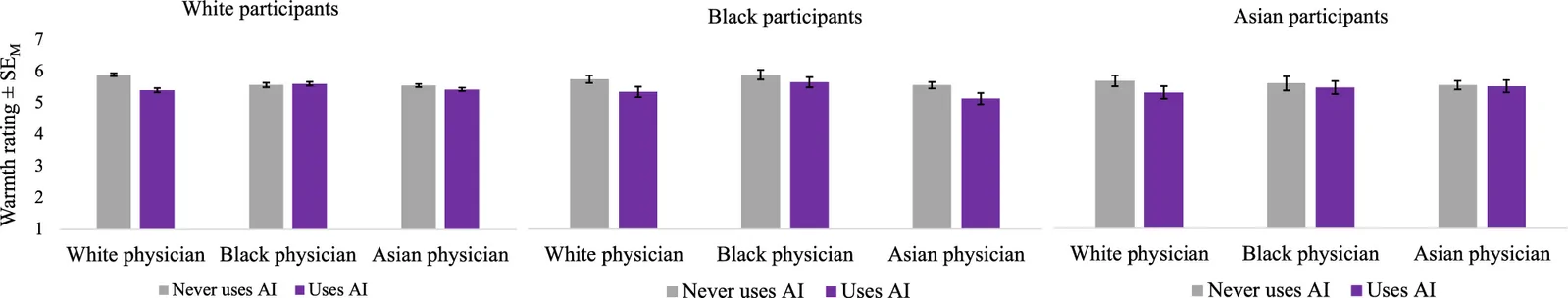
Interaction of consumer and physician race. AI-use penalties for perceived warmth differed between participant-physician pairs. Error bars show standard errors of the individual means.

We conducted exploratory (not preregistered) analyses examining three-way interactions between AI-use condition, physician, and participant demographics. As shown in Fig. 2, stated AI-use had a more negative impact among younger participants (<45 years old) across all measures, *F*s ≥ 5.82, *p*s ≤ 0.016, except for the likelihood to request a second opinion from another doctor, *F* < 1. For perceived warmth, there was a significant three-way interaction of AI-use, physician race, and participant race (see Fig. 3), F(4, 1698) = 2.47, *p* = 0.044, *η*_*p*_^2^ = 0.01 (Greenhouse-Geisser corrected). The negative effect of AI use was strongest for White physicians among White and Asian participants, and for Asian physicians among Black participants.

Overall, physicians who stated that they use (vs. never use) AI were perceived as significantly less warm and competent. This AI-use penalty manifested also in various behavioral intentions, like a reduced appointment willingness. Thereby, we extend prior research in this context (e.g., ref. ^4^), which focused on a limited set of evaluation criteria (e.g., trustworthiness). Moreover, our findings shed light on the moderating influence of physician and consumer demographics. Importantly, the observed penalty was largely insensitive to physician demographics (age, gender, race), suggesting that AI-use functions as a salient cue. However, exploratory analyses suggest that AI-use penalties are more pronounced for younger participants and vary, at least in part, across physician–consumer combinations.

A cue-based perspective (e.g., TrAM; ref. ^1^) helps explain these findings. In the absence of direct information about a physician’s ability or intentions, consumers rely on observable signals. AI-use disclosure appears to function as such a cue, potentially being interpreted as a sign of “outsourcing” key aspects of clinical judgment. Specifically, AI use may signal a lack of benign intent (warmth) or expertise (competence).

Importantly, our exploratory analyses suggest that at least certain consumer demographics may influence the perception of AI use, with larger evaluation penalties for participants below 45 years. Younger participants, who may be more familiar with AI (see e.g., ref. ^12^), could also be more aware of respective limitations and challenges, making them more skeptical regarding the implementation of AI in sensitive contexts like healthcare (see also ref. ^13^). Due to the exploratory nature of this analysis, these findings however should be interpreted with some caution and await confirmation in future research.

Furthermore, we found that specifically for White and Asian participants, the negative effect of AI-use on perceived warmth was most pronounced when evaluating White physicians, whereas for Black participants, the strongest warmth penalty emerged when evaluating Asian physicians. While this analysis was not preregistered and could to some extent be driven by the within-subject manipulation of physician race, this pattern suggests that the social consequences of AI use in healthcare may depend not only on physician or consumer characteristics in isolation, but also on their combination. Even though this effect was relatively small and we did not directly measure the underlying mechanism, AI use may thereby magnify existing asymmetries in trust, expectations, and perceived relational fit between consumers and physicians from different backgrounds.

These findings highlight that adopting AI is not merely a technical or organizational challenge but also a social one (see also ref. ^14^), which should be considered alongside performance and efficiency gains when evaluating AI adoption. Second, these results underscore the importance of how AI use is communicated and enacted in consumer interactions (see also ref. ^15^). Different ways of introducing and explaining the use of AI—emphasizing physician responsibility, shared decision-making, and consumer benefit—might mitigate negative perceptions. Third, training programs for healthcare professionals using AI may require communication strategies that are specifically designed to address consumer concerns about AI (see also ref. ^16^). Our results suggest that these communication strategies need to be tailored to the unique situation. For instance, addressing concerns toward the use of AI may be particularly important for younger consumers. Given the limitations of current AI systems, this however should not be understood as a call for blind trust in AI and a healthy skepticism toward AI in healthcare is certainly appropriate.

The external validity of our findings is limited by the vignette-based study design, the salient manipulation of AI use, the focus on a primary-care setting and the recruitment of a Prolific sample (e.g., higher digital literacy; participants indicated relative positive attitudes toward AI). Furthermore, in actual practice, consumers are likely to encounter physicians in richer informational contexts, which may attenuate the relative weight of AI disclosure. Finally, vignettes may have differed in some minor, additional factors (e.g., lighting) and we did not test potential mechanisms underlying the observed AI-use penalty.

## Methods

### Sample

We collected the study data via Prolific (www.prolific.com) in November 2025 and we used Prolific’s “representative sample” feature to approximate the composition of the US adult population (based on the 2021 US census). Therefore, the sample was stratified across brackets for age (18–24, 25–34, 35–44, 45–54, and 55+), gender (male and female), and race (White, Mixed, Asian, Black, and Other), based on the demographics which participants reported when registering at Prolific. An a priori power analysis resulted in a required sample size of 800 participants (100 participants per group; 1-β = 80% for *d* = 0.4, *α* = 0.05; 2-sided testing; calculated with the *power.t.test* function in the *statistics* package of R version 4.1.1; based on the pairwise comparisons between both AI-use conditions). To account for dropout and uneven group sizes due to random group allocation, we recruited 1200 participants (roughly 150 per group). Datasets of participants who failed at least one of the manipulation checks at the end of the experiment were excluded from the analysis (*n* = 170, 14%; see Table S5 in the supplement for detailed exclusion rates for each experimental condition and manipulation check). The proportion of excluded participants may be partly attributable to the use of three separate manipulation checks (with failure on any single check leading to exclusion), as well as their placement at the end of the study, after the main measures had been completed. We replicated our analyses on the full sample without exclusions of participants who failed one of the manipulation checks (reported in the supplement). The results were virtually identical, leading to the same conclusions as those reported in the manuscript. These exclusions resulted in a final sample size of 1,030 participants (self-reported gender identities: 520 females, 487 males, 18 non-binaries and 5 participants preferred to not disclose their gender; self-reported age: *M* = 46.9 years, SD = 16.5 years; self-reported race: 123 Black, 72 Asian, 96 Hispanic, 11 Native American, 674 White, 47 other, 7 prefer not to say). A sensitivity analysis showed that this sample size provides a power of 80% to detect even small main and interaction effects of *f* ≥ 0.05 (*α* = 0.05; ANOVA with 8 groups and 3 measurements; calculated in G*Power 3.1).

More than half of the sample indicated a university degree as their highest level of education (7 no formal qualification, 15 secondary education, 366 high school, 440 bachelor, 151 master, 42 PhD, 9 prefer not to say). We preregistered our hypotheses, the analysis plan, and the targeted sample size before data collection had started (https://osf.io/e8hfr). All participants provided informed consent. The study was conducted in accordance with the Declaration of Helsinki, and the study protocol was approved by the ethics committee of Judge Business School at the University of Cambridge (approval number: 24-28).

### Experimental design and procedure

We employed a 2 × 2 × 2 × 3 design with physician gender (female vs. male), age (younger vs. older) and AI-use (uses AI vs. never uses AI) as between-subject factors and physician race (White vs. Black vs. Asian) as a within-subject factor. We manipulated AI use, physician gender, and age between-subjects to minimize demand characteristics and socially desirable responding. Physician race was manipulated within-subjects to maintain a feasible sample size, as a fully between-subjects design would have required substantially more participants. To minimize carryover and contrast effects, the order of physician profiles was randomized across participants. Furthermore, in contrast to prior work^4^, we used a “never uses AI” condition as our control group instead of not presenting any statement on AI use. This was done to establish a precise, unambiguous baseline of no AI involvement and to eliminate potential variations in participants’ baseline assumptions about the use of AI.

All participants were instructed to review three advertisements for family doctors (one advertisement for each race condition in randomized order, i.e., White vs. Black vs. Asian physician). Furthermore, regarding our between-subject factors, each participant was randomly assigned to one of eight groups, differing in terms of gender (female vs. male), age (younger vs. older), and AI use (uses AI vs. never uses AI) of the presented physicians. To emphasize real-world implications, we framed the task as a market research study that aims to determine the most suitable advertisement for a medical practice. Physician images in the vignettes were created with Google’s Gemini 2.5 Flash Image (respective prompts are provided in the supplement). While we took great care to create highly realistic images, it cannot be excluded that some participants noticed that images were AI-generated, which may have influenced their behavior in the experiment. Furthermore, we aimed to keep the images as similar as possible while allowing some minor variations (e.g., presence vs. absence of a second person in the background). This was intended to avoid presenting identical images across vignettes and to maintain realism, as the study was framed as a market research task in which participants evaluated multiple advertisements to identify the most suitable one for a medical practice. Due to the US sample, physician names were chosen to reflect typical naming conventions for the respective race (and gender) in the US (e.g., common American first names combined with racially distinctive last names) in order to maintain realism while minimizing potential confounds related to perceived language proficiency or cultural familiarity. At the same time, the use of racially distinctive names may have introduced additional differences between conditions next to our intended manipulations. All vignettes are presented in the OSF repository (https://osf.io/8ufmk). To ensure attention, each advertisement was presented for 20 s before the respective questions appeared. First, we assessed, using established scales, how warm and competent the presented physician is perceived, which constitute two fundamental dimensions of social perception^11^. We assessed perceived warmth via 7 items (trustworthiness, empathy, warmth, friendliness, well intentions, good nature, sincerity; Cronbach’s alpha = 0.97) and perceived competence via 6 items (competence, confidence, capability, efficiency, intelligence, skillfulness; Cronbach’s alpha = 0.97). Here, trustworthiness was included as one indicator of warmth, consistent with prior operationalizations of the stereotype content model^11^. This treatment reflects our focus on patients’ broader, stereotype-based social evaluations of physicians. It differs from interpersonal trust models (e.g., ref. ^1^), in which trustworthiness is conceptualized as a superordinate construct comprising competence and warmth. We adopted the former, narrower use of trustworthiness as an indicator of warmth to remain consistent with the warmth-competence framework guiding our study. Next to warmth and competence, participants indicated their level of agreement with five statements (whether they would choose this doctor for their next appointment; whether they would recommend this doctor to a close friend or family member; whether they would follow this doctor’s treatment plan; whether after one visit, they would book with this doctor again; whether they would seek a second opinion after receiving a diagnosis or treatment plan from this doctor). For each statement, they made their response on a 7-point Likert scale (1 = “strongly disagree”, 7 = “strongly agree”). Finally, we asked participants how many minutes they would travel to see this doctor, how much they would pay for a consultation and how many days they would wait for an appointment (responses entered as numbers). Because these judgments require knowledge of typical values that participants may not have (e.g., average waiting time for a physician appointment in the US), we provided respective reference values. These reference values were identical across all conditions. While reference values may have biased the responses, any influence on absolute response levels is therefore constant across conditions and cannot account for between-condition differences, which should only reflect the experimental manipulation. Participants answered all questions for each advertisement separately.

At the end of the study we assessed participants use of AI-based tools (response options: never, rarely, occasionally, frequently, very frequently), their perception of the increasing integration of AI in healthcare (response options: more risks, neutral, more opportunities) and their view on the impact of AI in healthcare (response options: no, minor, moderate, significant, highly significant). Furthermore, we collected demographics (gender, age, race, educational level), queried participants’ patient status (“Based on your current health status, would you describe yourself as a patient?”; response options: yes, no, prefer not to say) and asked whether they received healthcare-related training or work in a healthcare-related profession (“Based on your training or current profession, would you describe yourself as a healthcare professional?”; response options: yes, no, prefer not to say). Finally, we implemented various manipulation checks. Participants should indicate the mentioned AI use (response options: “We use AI tools for diagnostics and therapy”, “We never use AI tools for diagnostics and therapy”, “There was no statement on AI tools in the advertisements”) as well as the assumed gender (response options: male, female, non-binary) and age (response options: 25–45 years, 45–65 years) of the presented physicians. After answering these questions, participants were debriefed about the study’s purpose.

### Data analyses

Data analyses were conducted in R (version 4.1.1). We computed composite indices for perceived warmth and competence by averaging the respective items. For the questions where participants should enter a numeric response (maximum travel time, payment, and waiting time), we excluded values that deviated more than three standard deviations from the respective cell mean. Please note that these exclusions were not preregistered but deemed necessary because of extremely high variance in these responses (e.g., one participant indicated a maximum travel time of >10,000 min). After applying this exclusion criterion, we additionally excluded two very strong outliers regarding the maximum travel time (average travel times of 167 min and 333 min with all remaining subject averages below 70 min). Results without these non-preregistered exclusions are reported in the supplementary information.

Next, we calculated a separate four-way analysis of variance (ANOVA) for each dependent variable (warmth, competence, willingness to make an appointment, willingness to recommend to a friend, treatment adherence, likelihood to book again, likelihood to request a second opinion, willingness to travel, willingness to pay, willingness to wait). For each ANOVA, the participant is treated as the repeated-measures unit. We included AI use (yes vs. no) as well as physician gender (female vs. male) and age (younger vs. older) as between-subjects factors and physician race (White vs. Black vs. Asian) as a within-subject factor. When Mauchly’s test indicated a violation of the sphericity assumption (the assumption that variances of the differences between all factor levels are equal), Greenhouse-Geisser correction was applied. This correction reduces the risk of inflated Type I error rates (false positives) that can arise when the sphericity assumption is violated. For all preregistered analyses (i.e., the four-way ANOVA for each of the 10 dependent variables), Benjamini-Hochberg procedure was used to adjust the threshold for statistical significance (baseline significance defined as 2-sided *p* < 0.05).

As an exploratory (not preregistered) analysis, we examined whether the effects of physicians’ AI use and demographic characteristics on evaluations also vary as a function of participant demographics. Therefore, we conducted an additional ANOVA for each measure, including participant age (≥45 vs. < 45 years old; we chose 45 years as the cut-off value based on typical definitions of “young adults”, see e.g., ref. ^17^), gender (female vs. male), and race (White vs. Black vs. Asian) as further between-subject factors (participants whose demographics did not match one of these categories were excluded from these analyses). For these exploratory analyses, no multiplicity adjustment was applied.

## Supplementary information

Supplement

## Data Availability

Underlying data are available in the OSF repository (https://osf.io/8ufmk).

## Code availability

The analysis script is available in the OSF repository (https://osf.io/8ufmk).
