# Supplementary material for: Influence of physician and consumer demographics on AI-use penalties in primary care: a vignette-based study
Source: NPJ Digit Med. 2026 Oct 1;9:743. doi: 10.1038/s41746-026-03361-3 (PMC13631233; doi:10.1038/s41746-026-03361-3)
Supplement: Supplementary file 1 [file 41746_2026_3361_MOESM1_ESM.pdf]

Supplement for

Influence of physician and consumer demographics on AI-use penalties in primary care: A vignette-based study

Moritz Reis, PhD[1]*; Jungmin Choi, PhD[2,3]; Yeun Joon Kim, PhD[2,3]; Yingyue Luna Luan, PhD[4]; Pengzhao Lyu, MSc[3]; Florian Reis, MD[5]

[1] Institute of Psychology, University of Wuerzburg, Wuerzburg, Germany

[2] Institute of Metabolic Science, School of Clinical Medicine, University of Cambridge, Cambridge CB2 0QQ, UK

[3] Cambridge Judge Business School, University of Cambridge, Cambridge CB2 1AG, Cambridge, UK

[4] UQ Business School, The University of Queensland, Brisbane, Queensland 4067, Australia

[5] Institute of Medical Informatics, Charité – Universitätsmedizin Berlin, Corporate Member of Freie Universität Berlin and Humboldt-Universität zu Berlin, Berlin, Germany

* Corresponding author. **Email:** moritz.reis@uni-wuerzburg.de

Content:

Sample characteristics

Stimuli generation process

Table S1-S4: Descriptive results

Table S5: Detailed exclusion rates

Results without data exclusions

Sample characteristics

Seventy-four participants (7%) indicated that they work in a healthcare-related profession and 569 participants (55%) described themselves as a patient. Private or work-related AI use varied substantially between participants ("never": *n* = 137, "less than once a month": *n* = 171, "at least once a month but not every week": *n* = 244, "at least once a week": *n* = 298, "at least once a day": *n* = 180). Participants also showed substantial variability in whether they perceived the increasing integration of AI into healthcare as presenting greater risks or opportunities. ("more risks than opportunities": n = 291; "more opportunities than risks": n = 467; "equally balanced risks and opportunities": n = 272). Finally, most participants believed that AI would have a remarkable impact on healthcare in the future ("no impact": *n* = 18, "minor impact": *n* = 53, "moderate": *n* = 270, "significant": *n* = 440, "highly significant": *n* = 249).

Stimuli generation process

We used the following prompt to generate the physician pictures in the vignettes (using Google's Gemini 2.5 Flash Image):

„Create an image of a male [female], White [Black / Asian], relatively old [young] physician. The physician should be prominently presented, and the image should be in portrait mode with a typical hospital hallway in the background. The image should be one as it might be encountered on a social media post or billboard. Show the following name on the name tag: Dr. [].“

Pictures were then inserted into our advertisement template, which was developed after reviewing various real-world advertisements for family doctors on social media. Because of the US sample and study context, all presented information (e.g., address and phone number) were adapted to US standards. The statement on AI-use ("We [never] use AI tools for diagnostics and therapy") was then added as a text box depending on the experimental condition.

Table S1-S4: Descriptive results

Table S1. Descriptive results for perceived warmth, competence and willingness to make an appointment.

Condition				Warmth		Competence		Appointment	
AI use	Age	Gender	Race	*M*	*SD*	*M*	*SD*	*M*	*SD*
no	older	female	White	5.83	1.12	5.82	1.17	5.08	1.48
no	older	female	Black	5.94	1.16	5.86	1.24	5.20	1.52
no	older	female	Asian	5.87	1.20	5.94	1.15	5.21	1.57
no	older	male	White	5.77	1.05	5.78	1.08	5.09	1.45
no	older	male	Black	5.95	1.08	5.92	1.14	5.36	1.48
no	older	male	Asian	5.87	0.98	6.03	0.95	5.30	1.38
no	younger	female	White	5.85	0.98	5.84	0.99	5.20	1.45
no	younger	female	Black	5.95	1.06	5.84	1.14	5.31	1.42
no	younger	female	Asian	5.86	1.03	5.92	1.08	5.28	1.43
no	younger	male	White	5.73	1.07	5.78	1.03	5.13	1.29
no	younger	male	Black	5.86	1.01	5.81	1.02	5.25	1.36
no	younger	male	Asian	5.74	1.10	5.94	0.99	5.26	1.35
yes	older	female	White	5.43	1.21	5.17	1.37	4.41	1.67
yes	older	female	Black	5.43	1.26	5.25	1.33	4.45	1.61
yes	older	female	Asian	5.45	1.20	5.42	1.32	4.53	1.72
yes	older	male	White	5.44	1.23	5.35	1.33	4.29	1.73
yes	older	male	Black	5.65	1.18	5.50	1.32	4.53	1.71
yes	older	male	Asian	5.41	1.33	5.47	1.36	4.41	1.67
yes	younger	female	White	5.29	1.32	5.12	1.57	4.29	1.82
yes	younger	female	Black	5.44	1.42	5.26	1.59	4.53	1.89
yes	younger	female	Asian	5.47	1.39	5.39	1.55	4.65	1.84
yes	younger	male	White	5.32	1.23	5.25	1.42	4.21	1.82
yes	younger	male	Black	5.47	1.28	5.28	1.48	4.32	1.86
yes	younger	male	Asian	5.40	1.23	5.36	1.50	4.37	1.90

Table S2. Descriptive results for willingness to recommend the doctor and adhere to this doctor's diagnosis or treatment plan.

Condition				Recommendation		Adherence	
AI use	Age	Gender	Race	*M*	*SD*	*M*	*SD*
no	older	female	White	4.88	1.54	5.47	1.43
no	older	female	Black	4.94	1.64	5.56	1.41
no	older	female	Asian	4.90	1.59	5.54	1.44
no	older	male	White	4.89	1.50	5.39	1.34
no	older	male	Black	5.03	1.58	5.67	1.30
no	older	male	Asian	5.04	1.45	5.69	1.24
no	younger	female	White	4.96	1.42	5.61	1.20
no	younger	female	Black	5.04	1.52	5.63	1.20
no	younger	female	Asian	5.01	1.45	5.66	1.29
no	younger	male	White	4.84	1.51	5.66	1.22
no	younger	male	Black	4.86	1.45	5.66	1.18
no	younger	male	Asian	4.82	1.50	5.64	1.28
yes	older	female	White	4.19	1.67	4.81	1.53
yes	older	female	Black	4.23	1.65	4.85	1.55
yes	older	female	Asian	4.41	1.68	4.95	1.54
yes	older	male	White	4.03	1.70	4.71	1.72
yes	older	male	Black	4.33	1.72	4.81	1.69
yes	older	male	Asian	4.16	1.62	4.82	1.68
yes	younger	female	White	4.12	1.80	4.78	1.69
yes	younger	female	Black	4.27	1.84	4.81	1.80
yes	younger	female	Asian	4.38	1.81	4.94	1.73
yes	younger	male	White	4.07	1.80	4.70	1.69
yes	younger	male	Black	4.09	1.86	4.78	1.69
yes	younger	male	Asian	4.23	1.88	4.84	1.74

Table S3. Descriptive results for willingness to book with this doctor again after one visit and likelihood to request a second opinion from another doctor.

**Condition**				**Book again**		**Second opinion**	
AI use	Age	Gender	Race	*M*	*SD*	*M*	*SD*
no	older	female	White	4.94	1.42	3.91	1.78
no	older	female	Black	5.00	1.54	3.84	1.85
no	older	female	Asian	5.03	1.58	3.72	1.85
no	older	male	White	5.02	1.45	3.88	1.77
no	older	male	Black	5.24	1.43	3.78	1.90
no	older	male	Asian	5.18	1.35	3.64	1.84
no	younger	female	White	5.21	1.27	3.85	1.92
no	younger	female	Black	5.15	1.34	3.63	1.82
no	younger	female	Asian	5.13	1.32	3.75	1.90
no	younger	male	White	5.11	1.30	4.05	1.72
no	younger	male	Black	5.15	1.27	4.08	1.71
no	younger	male	Asian	5.08	1.31	4.13	1.76
yes	older	female	White	4.33	1.50	4.29	1.71
yes	older	female	Black	4.47	1.56	4.20	1.68
yes	older	female	Asian	4.56	1.56	4.32	1.72
yes	older	male	White	4.15	1.67	4.34	1.78
yes	older	male	Black	4.55	1.62	4.43	1.79
yes	older	male	Asian	4.39	1.66	4.41	1.73
yes	younger	female	White	4.29	1.75	4.64	1.77
yes	younger	female	Black	4.40	1.76	4.52	1.74
yes	younger	female	Asian	4.58	1.77	4.42	1.81
yes	younger	male	White	4.15	1.74	4.38	1.84
yes	younger	male	Black	4.26	1.79	4.51	1.78
yes	younger	male	Asian	4.25	1.81	4.43	1.84

Table S4. Descriptive results for willingness to pay, travel and wait for an appointment with the presented doctor.

**Condition**				**Willingness to…**					
				pay [$]		travel [min]		wait [days]	
AI use	Age	Gender	Race	*M*	*SD*	*M*	*SD*	*M*	*SD*
no	older	female	White	76.33	41.89	28.57	13.47	13.55	9.08
no	older	female	Black	76.78	42.39	28.48	13.84	13.77	9.17
no	older	female	Asian	78.47	43.27	28.40	14.12	14.21	9.27
no	older	male	White	70.40	36.64	27.29	13.08	14.41	9.74
no	older	male	Black	73.70	36.28	28.00	13.16	15.58	10.72
no	older	male	Asian	73.64	36.24	28.65	12.10	15.88	10.23
no	younger	female	White	77.72	46.04	27.98	12.02	15.87	9.95
no	younger	female	Black	78.53	46.08	28.08	12.18	15.13	9.81
no	younger	female	Asian	78.78	46.33	28.81	12.58	14.97	9.19
no	younger	male	White	76.31	40.18	28.08	11.11	15.34	10.53
no	younger	male	Black	76.68	40.17	28.36	11.32	15.30	10.41
no	younger	male	Asian	78.25	40.87	28.72	10.95	15.44	10.27
yes	older	female	White	64.87	37.08	24.69	12.50	11.68	9.54
yes	older	female	Black	66.88	38.90	24.82	13.18	11.88	9.42
yes	older	female	Asian	65.99	37.80	25.45	13.61	12.29	10.06
yes	older	male	White	69.11	40.51	23.46	13.00	12.63	9.42
yes	older	male	Black	73.04	41.05	24.49	12.48	13.64	9.41
yes	older	male	Asian	71.90	41.63	24.60	13.13	13.41	9.81
yes	younger	female	White	66.29	43.52	22.59	13.61	10.81	9.46
yes	younger	female	Black	64.67	44.70	21.79	13.05	11.54	10.79
yes	younger	female	Asian	69.42	46.53	23.47	13.78	12.08	11.17
yes	younger	male	White	67.43	39.91	24.03	14.82	12.31	10.09
yes	younger	male	Black	66.70	40.59	23.63	14.44	11.85	10.04
yes	younger	male	Asian	67.55	41.06	24.41	14.38	12.20	10.11

Detailed exclusion rates

Table S5. Exclusion rates for each experimental condition and manipulation check.

Condition				Manipulation check			
AI use	Gender	Age	*N*	AI use	Gender	Age	Failed at least one
no	male	young	157	13%	4%	3%	17%
no	male	old	147	9%	4%	4%	12%
no	female	young	149	10%	3%	1%	12%
no	female	old	143	6%	3%	6%	13%
yes	male	young	157	11%	5%	3%	15%
yes	male	old	127	6%	4%	4%	12%
yes	female	young	169	13%	6%	4%	18%
yes	female	old	151	9%	4%	5%	15%
	Overall		1200	10%	4%	4%	14%

Results without data exclusions

We repeated our main analysis without excluding participants who failed one of the manipulation checks. In line with our previous analysis, participants evaluated AI-using physicians as significantly less warm, $F(1, 1192) = 41.56$, $p < .001$, $\eta_p^2 = .03$, and less competent, $F(1, 1192) = 55.76$, $p < .001$, $\eta_p^2 = .04$. Furthermore, participants in this condition were significantly less willing to make an appointment, $F(1, 1192) = 73.78$, $p < .001$, $\eta_p^2 = .06$, to recommend this doctor to a friend or relative, $F(1, 1192) = 56.08$, $p < .001$, $\eta_p^2 = .04$, to adhere to this doctor's treatment plan, $F(1, 1192) = 77.45$, $p < .001$, $\eta_p^2 = .06$, or to book with this doctor again, $F(1, 1192) = 66.11$, $p < .001$, $\eta_p^2 = .05$. Also, participants in this condition were significantly more likely to request a second opinion from another doctor, $F(1, 1192) = 30.98$, $p < .001$, $\eta_p^2 = .03$. Finally, participants reported that they would travel less far, $F(1, 1174) = 32.34$, $p < .001$, $\eta_p^2 = .03$, pay less money, $F(1, 1171) = 12.91$, $p < .001$, $\eta_p^2 = .01$, and wait less long for an appointment with an AI-using doctor, $F(1, 1175) = 21.66$, $p < .001$, $\eta_p^2 = .02$. Also, in line with our main analysis, neither of these effects significantly interacted with physician characteristics (age, gender, race) after applying Benjamini-Hochberg correction, Fs ≤ 4.31, ps $\geq .014$ (unadjusted p-value).

Finally, we also repeated our main analysis regarding willingness to travel, pay and wait, without excluding outliers. In accordance with the results reported in the main text, participants in the AI condition, indicated that they would pay significantly less money, $F(1, 1022) = 11.37$, $p = .001$, $\eta_p^2 = .01$, and would wait significantly less long for an appointment, $F(1, 1022) = 3.89$, $p = .049$, $\eta_p^2 < .01$. However, in this case, willingness to travel did not differ between AI-use conditions, $F < 1$. Again, there was no significant interaction of the AI-use condition and physician characteristics for any of the three dimensions, Fs ≤ 2.96, ps $\geq .054$.
